# Major Depression and Acute Coronary Syndrome-Related Factors

**DOI:** 10.5935/abc.20170028

**Published:** 2017-03

**Authors:** Jose Henrique Cunha Figueiredo, Nelson Albuquerque de Souza e Silva, Basilio de Bragança Pereira, Glaucia Maria Moraes de Oliveira

**Affiliations:** 1 Programa de Pós Graduação em Medicina (Cardiologia) - Universidade Federal do Rio de Janeiro, Rio de Janeiro, RJ - Brazil; 2 Instituto do Coração Edson Saad - Universidade Federal do Rio de Janeiro, Rio de Janeiro, RJ - Brazil

**Keywords:** Acute Coronary Syndrome, Depressive Disorder, Major, Social Class, Life Style

## Abstract

**Background:**

Major Depressive Disorder (MDD) is one of the most common mental illnesses in psychiatry, being considered a risk factor for Acute Coronary Syndrome (ACS).

**Objective:**

To assess the prevalence of MDD in ACS patients, as well as to analyze associated factors through the interdependence of sociodemographic, lifestyle and clinical variables.

**Methods:**

Observational, descriptive, cross-sectional, case-series study conducted on patients hospitalized consecutively at the coronary units of three public hospitals in the city of Rio de Janeiro over a 24-month period. All participants answered a standardized questionnaire requesting sociodemographic, lifestyle and clinical data, as well as a structured diagnostic interview for the DSM-IV regarding ongoing major depressive episodes. A general log-linear model of multivariate analysis was employed to assess association and interdependence with a significance level of 5%.

**Results:**

Analysis of 356 patients (229 men), with an average and median age of 60 years (SD ± 11.42, 27-89). We found an MDD point prevalence of 23%, and a significant association between MDD and gender, marital status, sedentary lifestyle, Killip classification, and MDD history. Controlling for gender, we found a statistically significant association between MDD and gender, age ≤ 60 years, sedentary lifestyle and MDD history. The log-linear model identified the variables MDD history, gender, sedentary lifestyle, and age ≤ 60 years as having the greatest association with MDD.

**Conclusion:**

Distinct approaches are required to diagnose and treat MDD in young women with ACS, history of MDD, sedentary lifestyle, and who are not in stable relationships.

## Introduction

Prior studies have attempted to understand the factors influencing the prognosis of an acute coronary event, and screening for symptoms of depression has been recommended as routine for acute coronary syndrome (ACS) patients.^1^ Although the association between depression and a worse prognosis in ACS patients has been documented in a number of studies,^2-4^ only recently did the American Heart Association recommend it be included as a risk factor for adverse ACS outcomes, even if they emphasize the heterogeneity of the studies employed in the systematic review on which the recommendation was based.^5^

The prevalence of depression in ACS patients in the USA was estimated at 20%, thus affecting 15.4 million adult coronary artery disease patients.^6^ In São Paulo, Brazil, a study using the Beck Depression Inventory reports symptoms of depression in 43.5% of the patients hospitalized with ACS.^7^ Another study^8^ found a similar prevalence rate, 46.7%, and concluded that women, men under 50 years of age and people suffering from anxiety are more likely to show signs of depression when screened for depression [*Primary Care Evaluation of Mental Disorders* (Prime MD) and BDI], trait anxiety and state anxiety (IDATE), and alcohol consumption (AUDIT). These rates depict the greater sensitivity of screening tools.

The Danish National Patient Registry, which gathered a cohort of around 83,000 ACS patients, pointed to excessive mortality in those with marked inequalities in education, even when adjusted for prior comorbidities and depression,^9^ raising the hypothesis that depression may fit into an adverse social context that is most common among women. Therefore, questions still remain as to the prevalence of depression where ACS is concerned, and its influence on prognosis and associated factors, especially in regard to the Brazilian population.

The aims of this study are to check the prevalence of Major Depressive Disorder (MDD) in patients diagnosed with ACS in three public hospitals in the city of Rio de Janeiro and analyze factors potentially associated with MDD within this setting.

## Methods

This is an epidemiological, observational, descriptive, cross-sectional study involving patients hospitalized with ACS who have been diagnosed using clinical, enzymatic and electrocardiographic criteria. The research protocol was submitted to and approved by the Research Ethics Committee, and the participants signed a Free and Informed Consent Form (HSE, nº 160/04).

We used the Acute Coronary Care (ACC) of three public hospitals used for both teaching and medical care in Rio de Janeiro City: a federal general hospital, a municipal general hospital, and a state hospital specializing in cardiology. The study was carried out over the course of 24 consecutive months. Together the three ACC had a total of 21 hospital beds.

Included in the study were male and female patients over the age of 20 admitted to the cardiology units of the three participating hospitals. Excluded were the patients clinically unable to answer the interview by their seventh day of hospitalization at the ACC, as well as patients clinically deemed unable to respond to the interview due to cognitive alterations or auditory deficiencies that would preclude an oral interview.

The interview and application of the research tools were carried out by the seventh day of hospitalization in the ACC. All the tools used were read by the patient and subsequently applied in a single interview by one of the authors. Patients responded to the Structured Clinical Interview of the *DSM-IV Axis I Disorders - Patient Edition (SCID-I/P, version 2.0)* (Appendix 1).^10-12^ For this study, we applied the section on Major Depressive Episodes (MDE),^13^ as well as a standardized questionnaire on sociodemographic, lifestyle and clinical data.

The sociodemographic variables contemplated were gender, age, marital status (married or in common-law marriages according to Brazilian law or living together in a stable relationship; not married when not living together in marital situation), level of education (years of schooling completed or not completed dichotomized into under four years or over four years), family income (monthly income of all family members sharing the overall common costs of living in three categories: group A, up to US 615 monthly wages; group B, between US 615 and US 1230 monthly wages; and group C, more than US 1230 monthly wages). Social support (two dimensions assessed: family and close friends) consolidated in two categories: patients who lived alone and/or had no friends were considered to have "no social support", and those who didn't live alone and did have friends were considered to "have social support".

The variables referring to lifestyle were tobacco smoking (a "smoker" is someone who reported smoking cigarettes up to one year prior to the current coronary event, and a "non-smoker" is someone who reported having quit smoking more than a year before the latest coronary event or who had never smoked before), and sedentary lifestyle ("sedentary" is someone who reported not practicing any regular physical activity - walking, jogging, riding a bicycle, practicing sports - for at least 30 minutes three times a week at the least).

The clinical variables found were dyslipidemia, hypertension and diabetes mellitus (when self-reported, with elevated or normal serum values associated with specific drugs used for treatment and confirmed on the medical records, and with increases in systolic and diastolic blood pressure in the case of hypertension); prior acute myocardial infarction (AMI - taken from the medical records); Killip class, dichotomized into Killip 1 and Killip ≥ 2; and ongoing major depression, verified by a structured interview (SCID-I/DSM-IV) to estimate point prevalence and throughout life.

The selection of variables was based on the association with coronary syndrome and observed in previous studies in non-Brazilian population.

### Statistical analysis

We employed the chi-squared test to assess dependence between sociodemographic, lifestyle and clinical variables and MDD, and the Mantel-Haenszel test to assess dependence between sociodemographic, lifestyle and clinical variables and MDD when controlled by the variable gender. In our analysis of the results, besides statistical significance (p < 0.05), we considered clinical significance (0.05 < p ≤ 0.15) to be a factor that explains the association in question. We used a general log-linear multivariate model of analysis to assess the level of association between variables of interest. The statistical program used was the R system, version 2.1.1.

## Results

This study assessed 356 patients (229 men), whose ages varied from 27 to 89 years. The average and median age was 60 (SD ± 11.42) years. Average age for the women was 62 years, and for the men, 59, suggesting that women tend to suffer ACS later in life. Average time hospitalized at the ACC was 9.7 days.

The point prevalence we identified for the current MDD, according to DSM-IV diagnosis criteria, was 23% (82 of the 356-patient sample group).

The sociodemographic, lifestyle and clinical characteristics categorized by presence of MDD are shown in Table 1.

**Table 1:** Sociodemographic, lifestyle and clinical characteristics according to the presence or absence of major depressive disorder (MDD)

	MDD n (%) 82 (23.0)	Non-MDD n (%) 274 (77.0)	Total (n) 356
	34 (14.8)	195 (85.2)	229
> 60 years	34 (19.5)	140 (80.5)	174
Married***	44 (19.5)	182 (80.5)	226
Schooling > 4 years	40 (21.1)	150 (78.9)	190
With social support	63 (21.8)	226 (78.2)	289
**Family income**			
A (≤ US 615 m.w.)	28 (27.5)	74 (72.5)	102
B (US 615-1230 m.w.)	27 (23.1)	90 (76.9)	117
C (> US 1230 m.w.)	27 (19.7)	110 (80.3)	137
Tobacco smoking	30 (24.4)	93 (75.6)	123
Sedentary lifestyle**	69 (26.3)	193 (73.7)	262
Dyslipidemia	40 (26.0)	114 (74.0)	154
SH	58 (23.1)	193 (76.9)	251
Diabetes	23 (22.8)	78 (77.2)	101
History AMI	20 (20.8)	76 (79.2)	96
History MDD	33 (53.2)	29 (46.8)	62
Killip ≥ 2	10 (37.0)	17 (63.0)	27

Pearson chi-squared test, p < 0.0001*, p < 0.01**, p < 0.05****, † clinical relevance. SH: systemic hypertension; m.w.: monthly wages; AMI: acute myocardial infarction.

The MDD prevalence was higher in the patients ≤ 60 years of age (26.4% x 19.5%), though not statistically relevant. Regarding marital status, 226 (63.5%) of the patients were married or in a stable relationship, 31 (8.7%) were single, 48 (13.5%) were separated, divorced or legally separated, 51 (14.3%) were widowed, and the MDD prevalence among the unmarried ones was statistically significant. There was a greater prevalence of MDD in those with *less than 4 years* of schooling and those without *social support*, but without statistical significance, perhaps due to the size of the sample group in question. As for family income, subgroup A counted 102 (28.7%) patients, of whom 28 (27.5%) were depressed; subgroup B had 117 (32.9%) patients, with 27 (19.7%) of them depressed; and subgroup C comprised 137 (38.7%) patients, with 27 (19.7%) of them found to be depressed. Though there was no statistical difference between the subgroups, what stands out is the progressive drop in the rate of depressed patients according to how high the family income was (Table 1).

The presence of depression was significantly greater among sedentary patients but not among smokers, dyslipidemic, diabetic or hypertensive patients, nor those with a history of AMI. The Killip class ≥ 2 was found to have the greatest prevalence of MDD, and was clinically relevant. There were 62 (17.4%) patients with a history of MDD, of whom 33 (53.2%) were found to be depressed, and that was statistically relevant (Table 1).

In summary, this initial analysis of the variables gender, marital status, sedentary lifestyle, Killip class ≥ 2 and history of MDD associated significantly with MDD in the ACS index event (Table 1).

Table 2 shows the results controlled for gender. The frequency of depressed females was significantly greater than that found for males (37.8% x 14.8%), with a three-and-a-half-time greater likelihood of developing MDD than the male subgroup. Regarding age, for both sexes, the odds ratio was less than 1, and older age was found to be more protective against depression; the data suggest that this protection is greater in men than in women.

**Table 2:** Sociodemographic, lifestyle and clinical characteristics according to the presence or absence of major depressive disorder (MDD) controlled for gender

	Female			Male		
Characteristics	MDD N (%)	Non-MDD N (%)	OR	MDD N (%)	Non-MDD N (%)	OR
Female gender^1^*	48 (37.8)	79 (62.2)		34 (14.8)	195 (85.2)	3.485
≤ 60 years^2^***	22 (40.7)	32 (59.3)	0.81	26 (20.3)	102 (79.7)	0.34
Unmarried^2^	30 (39.0)	47 (61.0)	0.88	8 (15.1)	45 (84.9)	0.98
Schooling > 4 years^2^	40 (38.5)	64 (61.5)	0.85	23 (12.4)	162 (87.6)	2.35
Family income > 6 m.w.^2^	13 (41.9)	18 (58.1)	0.84	14 (13.2)	92 (86.8)	1.57
Smoker^2^	13 (39.4)	20 (60.6)	0.91	17 (18.9)	73 (81.1)	0.6
Sedentary lifestyle^2†^	41 (38.7)	65 (61.3)	0.79	28 (17.9)	128 (82.1)	0.41
Dyslipidemia	28 (39.4)	43 (60.6)	0.85	12 (14.5)	71 (85.5)	1.05
SH^2^	38 (36.9)	65 (63.1)	1.22	20 (13.5)	128 (86.5)	1.34
Diabetes^2^	15 (34.1)	29 (65.9)	1.28	8 (14.0)	49 (86.0)	1.09
Killip ≥ 2^2^	6 (42.9)	8 (57.1)	0.81	4 (30.8)	9 (69.2)	0.36
History AMI^2^	13 (35.1)	24 (64.9)	1.18	7 (11.9)	52 (88.1)	1.4
History MDD^2^*	20 (64.5)	11 (35.5)	0.23	13 (41.9)	18 (58.1)	0.16

1 Chi-squared test,

2 Mantel-Haenszel test, p < 0.0001*, p < 0.01**, p < 0.05***,

† clinical relevance. SH: systemic hypertension; AMI: acute myocardial infarction; OR: odds ratio; m.w.: monthly wage.

Depression was found at a greater frequency in women than in unmarried men. Despite percentage differences, type of marital relationship did not relate significantly to MDD when controlled for gender. We also found that, percentage-wise in this sample, women have lower levels of education than men, and those with less schooling had a greater likelihood, albeit not by much, of becoming depressed than those with more schooling. Among men there was no difference in this respect. Among women we found practically no difference between the with- or without-social support categories. In contrast, we found that men without social support were nearly two and a half times more likely to become depressed than those with social support. However, there was no statistical difference between genders when comparing the variable social support (Table 2).

Taking as a reference family income in subgroup A, we found that women in this subgroup are more likely to show signs of depression than those in subgroup B, and less likely than those in subgroup C. Among the men, those in subgroup A were more likely to become depressed than those in subgroup B and subgroup C. However, between men and women the odds ratio of subgroups B and C compared to subgroup A was not significant (Table 2).

Regarding smoking, the women had a much higher rate of depression than the men. Nevertheless, what became clear was that the likelihood of male smokers becoming depressed was greater than that of non-smokers, which was not the case with the women. The results pointed to a greater number of sedentary persons of both sexes, with the frequency among women being greater than among men. Sedentary persons were found to have greater odds of becoming depressed regardless of their gender. However, the sedentary men were around 2.5 times more likely to develop MDD than the non-sedentary ones. Unlike the men, the chance of a woman becoming depressed while sedentary was much lower (Table 2).

There appears to be a greater tendency toward becoming depressed among males, however slight, where having or not having a history of hypertension is concerned. We found that non-diabetic women were slightly more likely to become depressed than those with diabetes. Among the men there was practically no difference between those with a history of diabetes and those without a history of diabetes. There was no statistically significant association between diabetes and MDD (Table 2).

Apparently, patients of either gender without a history of AMI had greater chances of becoming depressed than those *with* a history of AMI. We also noted that there were greater odds for men than for women. However, we did not find a statistically significant association with MDD. Regardless of gender, patients without a history of MDD have greater protection against depression in the index event (Table 2).

In summary, when controlled for gender, the association between MDD and the variables age ≤ 60 years, sedentary lifestyle and history of MDD is statistically significant (Table 2).

**Figure 1:**
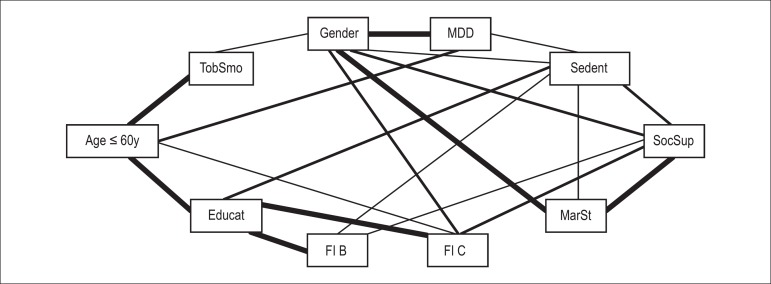
Graphical representation of the general log-linear model demonstrating the interdependent relationships between the sociodemographic variables and the powers of association without history of major depressive disorder (MDD). Sedent: sedentary lifestyle; SocSup: social support; MarSt: marital status; FI B: family income B (US 615-1230 monthly wages); FI C: family income C (> US 1230 monthly wages); Educat: education level; TobSmo: tobacco smoking.

**Figure 2:**
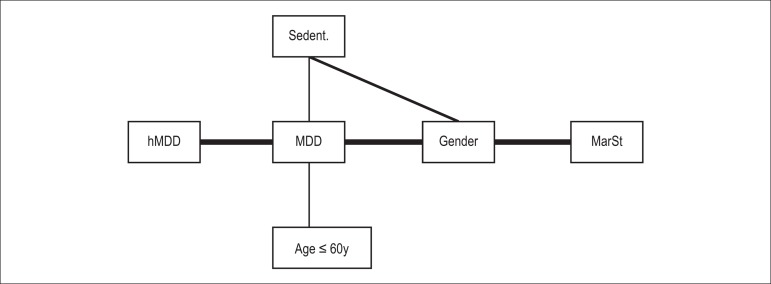
Graphical representation of the general log-linear model demonstrating the interdependent relationships between the variables examined and the powers of association. MDD: major depressive disorder; MarSt: marital status; Sedent.: sedentary lifestyle; hMDD: history of MDD.

To assess the power of association in the general log-linear model, we chose to represent it in Figures 1 and 2, with a thick line that thins as the power of association progressively decreases according to estimates of (λ's) parameters of the log-linear model.

To perform the multivariate analysis we used the log-linear model that allows us to assess the associations of all the variables all together. We found direct relationship between the variable MDD and gender, age ≤ 60 years and marital status. Other variables were related to MDD conditioned by one or more of the variables directly related to MDD (Figure 1). (Appendix 2)

We also found that the power of association was greater (thicker line) between MDD and history of MDD (λ = 17.387) and MDD and gender (λ = -11.755), and was weaker (thinner line) between MDD and sedentary lifestyle (λ = 0.6026), and much weaker (thin line) between MDD and age ≤ 60 (λ = 0.3886). Gender and marital status (λ = -16.320) associated strongly (thicker line), while gender and sedentary lifestyle associated less strongly (thinner line) (λ = 0.7402). The association between MDD and marital status was conditioned to gender (Figure 2).

## Discussion

In this study, 23% of the patients with ACS met the criteria for MDD. This evidence in coronary-patient sample groups is similar to that reported in the international literature, and has prognostic implications that show a rise in mortality from all causes, and cardiovascular mortality between 12 months to 5 years after the ACS index event, even when evaluating MDD.^14-17^ It is noteworthy that in this study the use of a diagnostic measuring instrument - DSM-IV clinical diagnostic interview - that differs from tracking scales, and the time criterion for diagnosing MDD was maintained, which means that all the patients who met the MDD/DSM-IV criteria were already depressed at the time of the coronary event. Thus, the MDD prevalence having been found to be much higher than that of the overall population could suggest that there are common factors shared between the development of MDD and ACS. One study^9^ pointed to social inequality, especially where education is concerned, and comorbidities as factors that are present in and associated with depression and ACS. On the other hand, MDD and ACS seem to share such inflammatory biomarkers as cytokines, alterations in oxidative stress, platelet alterations, and vascular reactivity, with an array of complex biological interactions that are so far not fully understood.^18-20^

The rate of depression in women has varied from one and a half time to three times that of men.^21,22^ This difference was found in this study, where the rate of MDD in men was 14.8% and in women, 37.8%. In the descriptive analysis controlling for gender, the women were at three-and-a-half-time greater risk of developing MDD than men. The reasons for women being more susceptible to MDD than men remain obscure, in spite of studies^23^ that found an association between the neuroticism factor of personality (moodiness, worry and nervousness) and the female gender and severer depression.

Also noteworthy is the fact that we found a greater rate of depression in patients of both genders ≤ 60 years of age, which converges with the findings from another study.^24^ One must remember that this stage of life is the most productive and is still early, in current terms, for the subjects to incur such subjective experiences as limitations or threat to their lives, or objective experiences that give rise to worries regarding socio-familial responsibilities, which make them more susceptible to depression.

Marital status influenced the rate of depression in patients with ACS.^25^ The general log-linear model also showed that there is an association between the variable marital status and MDD conditioned to gender. What can be inferred from this is that this association pertained to the unmarried and females, who, as aforementioned, showed a greater tendency to MDD than married persons and males.

Regarding level of schooling, what needs to be put into perspective is that this sample group, having come from public hospitals and thus tending to be from lower socioeconomic classes, was expected to have a lower level of schooling, which proved to be the case. Females had a lower level of schooling than did men, and those from the less schooled subgroup were slightly more likely to become depressed than those with higher levels of schooling, which was not the case with men. Less formal education among females is congruent with this generation who had less opportunity to study than to focus on their families.

Mankind, being essentially gregarious by nature, needs the company of others who would generally comprise part of a social support network. Falling ill and being hospitalized produce suffering, isolation and a feeling of solitude from being away from home, work, friends and family. This social support network is relevant because it is within it that the sick and hospitalized individual, oftentimes, seeks emotional support. There was an association between perceived social support and lower cardiovascular reactivity in depression and ACS sufferers,^26^ with a decrease in cardiac mortality.^20,27^ In this study, we found that patients without social support tended to become depressed more often that those with it, though the difference was not statistically significant. When the analysis was controlled for gender, we found that this tendency held true for men without social support, who were two and a half times more likely to become depressed than those with social support.

The rates of depression by income were similar to those found in the global sample, although it dropped from 27.5% to 19.7% as family income increased, suggesting that the lower-income strata of society have a greater tendency toward depression, though the difference is not statistically significant. When the analysis was controlled for gender, the female subgroup was found to change the order that was found without this control, and the much higher rates of depression remained stable in the lower-income and intermediary socioeconomic classes (37.8% and 35.3%), only slightly rising in the highest family-income class (41.9%). In the male subgroup the rates were much lower and inversely related to the female subgroup, having decreased (19.3%, 13.6% and 13.2%), without a statistically significant difference. The women of the highest income class tended to become depressed more often, though only slightly, unlike the men, probably because the causes of depression relating to this variable are distinct between genders.^28^

In the overall sample, we found higher rates of depression among sedentary persons than non-sedentary ones, with a statistically significant association. When the sample was controlled for gender, the statistical significance dropped, but the association remained relevant because of the male subgroup, where the sedentary men were around 2.5 times more likely to become depressed than the non-sedentary ones. This is understandable, as the symptoms of depression could explain the higher rate of sedentary lifestyle among depressed men.^29^

The significant association found between patients with MDD and with and without a history of MDD corroborates the findings from other studies.^1,2,4,22,30^ This finding was not surprising, because a 50% rate of MDD relapse is expected to follow the initial episode, regardless of gender. By the same token, we found that having a history of MDD reflected in the female subgroup with around four-and-a-half-time greater likelihood of becoming depressed, and in the male subgroup with around six-time higher chance of becoming depressed than those who had never had MDD - a finding of major clinical importance.

### Clinical implications

The highlight of this multivariate analysis was the capacity to evaluate the interdependence of various indistinct variables; that is, the fact that all the variables are response variables concedes to them the same importance and increases the likelihood of their being applied in clinical practice. Our findings point to a need for a distinct approach to diagnosing and treating MDD in female ACS patients, ≤ 60 years of age and with a history of MDD, sedentary lifestyle and who are not in a stable marital relationship. The prognostic implications of these findings need further analysis in future studies.

### Limitations

If on the one hand the descriptive statistics controlling for gender were significant, on the other they were insignificant in the analysis of the subgroups for having produced results that must be considered with caution. The fact that the study was carried out in public hospitals precludes the possibility of generalizing the data, even though around 70% of the Brazilian population is treated in the Brazilian public healthcare system.

Although there are no Brazilian studies on the subject using an interview considered gold standard for the diagnosis of major depression, the authors recognize the sample size as small.

## Conclusion

This study found a 23% prevalence of patients with ACS meeting the diagnostic criteria for MDD. Females were more susceptible to developing MDD in the sample group of ACS patients, with a three-and-a-half-time greater likelihood than males.

Social support, sedentary lifestyle and Killip class ≥ 2 were variables that directly related to the male gender, the subgroup being around two-and-a-half-time more likely to develop MDD than the female subgroup.

History of MDD, regardless of gender, strongly associated with the current MDD, with the chances for women being a little more than four times, and for men, around six times.

The general log-linear multivariate analysis suggests that history of MDD, gender, sedentary lifestyle and age ≤ 60 years are the variables with the greatest power of association with MDD in this sample of ACS patients.

## APPENDIX 1

**Table:** Structured Clinical Interview for DSM-IV (SCID-DSM IV) - Criteria for Major Depressive Episode.

A) At least 5 of the following were present (+) during the same period of 2 weeks, representing a change in the previous functioning; at least 1 symptom is (1) depressed mood, or (2) loss of interest or pleasure.

In the last month...
(1) ... has there been a period of time when you were feeling depressed or down most of the day, nearly every day? How was that? If yes, how long did it last? (For as long as 2 weeks?)
(2) ... have you lost interest in things that you would usually enjoy? If yes, was it nearly every day? How long did it last? (For as long as 2 weeks?)
[For 2 weeks]...
(3) ... have you lost or gained weight? How much? Were you trying to lose weight? If not, how was your appetite? And how do you compare with your usual appetite? Have you forced yourself to eat? Have you eaten more/less than usual? Has this happened nearly every day?
(4) ... how have you been sleeping? Trouble falling asleep, waking frequently in the night or early morning, or have you been sleeping more than usual? How many hours per night, as compared to usual? Has this happened nearly every day?
(5) ... have you been feeling so fidgety or restless, that you feel unable to sit still? Was it so intense that people around you would notice? What did they notice? Has this happened nearly every day? If not, on the contrary - have you spoken or moved more slowly than usual? Was it so intense that people around you would notice? What did they notice? Has this happened nearly every day?
(6) ... how was your energy level? Have you felt fatigued all the time? Nearly every day?
(7) ... how have you been feeling about yourself? Worthless? Nearly every day? If not, have you felt guilty about things you have or have not done? Nearly every day?
(8) ... have you had problems concentrating or thinking? Has that interfered with anything? Nearly every day? If not, has it been hard to make decisions about everyday things?
(9) ... things were so bad that you had thoughts of dying, or that it would be better to die? And what about killing yourself? If yes, have you ever tried to kill yourself

## APPENDIX 2

**Table:** Results of the log-linear model to assess the relationship between the sociodemographic variables, including the variable major depressive disorder (MDD)

	Estimator (λ)	Standard error	Z Value	Pr (>|z|)	
(Intercept)	-2.688.449	0.635332	-4.232	2.32e-05	***
MDD	-0.821252	0.548862	-1.496	0.134580	
Gender	2.251.472	0.492674	4.570	4.88e-06	***
Age ≤ 60	-1.172.157	0.513623	-2.282	0.022481	*
Marital status	1.795.698	0.479341	3.746	0.000180	***
Schooling	-1.422.957	0.521306	-2.730	0.006341	**
Social support	1.885.126	0.521265	3.616	0.000299	***
Family income (B)	0.180327	0.552230	0.327	0.744013	
Family income (C)	-1.249.277	0.602538	-2.073	0.038139	*
Smoking	-2.349.186	0.556843	-4.219	2.46e-05	***
Sedentary lifestyle	1.277.152	0.508897	2.510	0.012085	*
MDD: Gender	-1.316.731	0.301854	-4.362	1.29e-05	***
MDD: Age ≤60	0.620317	0.303374	2.045	0.040882	*
MDD: Marital status	-0.046769	0.305908	-0.153	0.878488	
MDD: Schooling	-0.165475	0.298806	-0.554	0.579724	
MDD: Social support	-0.477188	0.349738	-1.364	0.172437	
MDD: Family income (B)	-0.058983	0.340688	-0.173	0.862550	
MDD: Family income (C)	0.007777	0.354681	0.022	0.982506	
MDD: Smoking	0.148600	0.303039	0.490	0.623873	
MDD: Sedentary lifestyle	0.612873	0.350552	1.748	0.080411	.
Gender: Age ≤ 60	0.357111	0.289478	1.234	0.217338	
Gender: Marital status	-1.782.421	0.282649	-6.306	2.86e-10	***
Gender: Schooling	0.173322	0.285969	0.606	0.544456	
Gender: Social support	-1.043.043	0.359433	-2.902	0.003709	**
Gender: Family income (B)	-0.185105	0.325247	-0.569	0.569273	
Gender: Family income (C)	0.755098	0.345023	2.189	0.028630	*
Gender: Smoking	0.470905	0.302531	1.557	0.119577	
Gender: Sedentary lifestyle	-0.483353	0.318571	-1.517	0.129203	
Age ≤ 60: Marital status	-0.332687	0.285541	-1.165	0.243974	
Age ≤ 60: Schooling	1.213.026	0.264028	4.594	4.34e-06	***
Age ≤ 60: Social support	-0.018670	0.330887	-0.056	0.955004	
Age ≤ 60: Family income (B)	-0.437650	0.320723	-1.365	0.172388	
Age ≤ 60: Family income (C)	-0.478175	0.328037	-1.458	0.144927	
Age ≤ 60: Smoking	1.667.095	0.268469	6.210	5.31e-10	***
Age ≤ 60: Sedentary lifestyle	0.174090	0.285680	0.609	0.542267	
Marital status: Schooling	0.236244	0.284772	0.830	0.406771	
Marital status: Social support	-1.853.831	0.332073	-5.583	2.37e-08	***
Marital status: Family income (B)	-0.253452	0.325323	-0.779	0.435933	
Marital status: Family income (C)	-0.321234	0.335701	-0.957	0.338614	
Marital status: Smoking	0.142201	0.293944	0.484	0.628551	
Marital status: Sedentary lifestyle	0.530015	0.312457	1.696	0.089833	.
Schooling: Social support	0.246803	0.331015	0.746	0.455912	
Schooling: Family income (B)	1.090.231	0.311334	3.502	0.000462	***
Schooling: Family income (C)	1.755.401	0.316313	5.550	2.86e-08	***
Schooling: Smoking	0.348653	0.275326	1.266	0.205395	
Schooling: Sedentary lifestyle	-0.741624	0.286969	-2.584	0.009757	**
Social support: Family income (B)	0.648041	0.362608	1.787	0.073911	.
Social support: Family income (C)	0.993385	0.388036	2.560	0.010466	*
Social support: Smoking	0.264417	0.344911	0.767	0.443305	
Social support: Sedentary lifestyle	0.709920	0.341477	2.079	0.037620	*
Family income (B): Smoking	-0.101796	0.332136	-0.306	0.759233	
Family income (C): Smoking	0.093087	0.328933	0.283	0.777179	
Family income (B): Sedentary lifestyle	-0.681732	0.354139	-1.925	0.054225	.
Family income (C): Sedentary lifestyle	-0.392269	0.359034	-1.093	0.274584	
Smoking: Sedentary lifestyle	-0.115416	0.285370	-0.404	0.685887	

**Table:** Family incomes (B) and (C) - categories B and C defined in this study.

	Estimator (λ)	Standard error	Z Value	Pr (>|z|)	
(Intercept)	10.180	0.2687	3.788	0.000152	***
MDD	-16.204	0.3859	-4.199	2.68e-05	***
Gender	21.191	0.2832	7.482	7.31e-14	***
Sedentary lifestyle	14.193	0.2590	5.479	4.27e-08	***
History of MDD	-21.340	0.1964	-10.867	< 2e-16	***
Marital status	0.4318	0.1816	2.377	0.017437	*
Age ≤ 60	-0.0438	0.1209	-0.362	0.717020	
MDD: Gender	-11.755	0.2639	-4.454	8.43e-06	***
MDD: Sedentary lifestyle	0.6026	0.3401	1.772	0.076453	.
MDD: History of MDD	17.387	0.2988	5.819	5.92e-09	***
Gender: Sedentary lifestyle	-0.7402	0.2850	-2.598	0.009390	**
Gender: Marital status	-16.320	0.2399	-6.804	1.02e-11	***
MDD: Age ≤ 60	0.3886	0.2547	1.526	0.126975	
